# Correlations of the triglyceride−glucose index and modified indices with arterial stiffness in overweight or obese adults

**DOI:** 10.3389/fendo.2024.1499120

**Published:** 2024-12-17

**Authors:** Yuchen Tang, Li Li, Jialin Li

**Affiliations:** Department of Endocrinology and Metabolism, The First Affiliated Hospital of Ningbo University, Ningbo, China

**Keywords:** arterial stiffness, insulin resistance, triglyceride-glucose index, modified TyG indices, obesity

## Abstract

**Background:**

Insulin resistance (IR) contributes substantially to the development of cardiovascular disease (CVD) and metabolic disorders, particularly obesity. The homeostatic model assessment of IR is a prevalent IR indicator, but insulin measurement is quite impractical for widely use. Given its convenience and accessibility, the triglyceride−glucose (TyG) index, along with modified indices such as the triglyceride−glucose−waist circumference (TyG−WC) and triglyceride−glucose−waist−height ratio (TyG−WHtR), are gaining recognition as practical tools for assessing IR. This study aimed to investigate the specific correlation between the TyG index and its modified indices with arterial stiffness in an overweight or obese population and to explore novel, self-defined modified TyG indices for identifying individuals at elevated risk for such conditions.

**Methods:**

This retrospective study included 1,143 overweight or obese individuals from 2021 to 2023. Medical data, including brachial-ankle pulse wave velocity (baPWV), were collected. Two novel modified TyG indices, TyG-1h and TyG-2h, were defined by substituting the fasting glucose level in the TyG formula with 1-hour and 2-hour post-load plasma glucose levels, respectively. Multivariate logistic regression analyses were conducted to identify parameters that demonstrated a statistically significant correlation with arterial stiffness, defined as a baPWV threshold of ≥ 1400 cm/s. Additionally, restricted cubic spline (RCS) modelling was employed to further explore these relationships in a visually interpretable manner. To evaluate and compare the diagnostic accuracy of the conventional TyG index and its novel modified versions, receiver operating characteristic (ROC) curve analyses were performed.

**Results:**

Our findings revealed that individuals with arterial stiffness presented significantly elevated TyG index and all its modified versions (P< 0.05). By utilizing a binary logistic regression model and adjusting for potential confounders, we determined that all TyG-related parameters independently correlated with an increased risk of developing arterial stiffness. Moreover, TyG-WHtR displayed the best correlation (OR 3.071, 95% CI 1.496-6.303) when stratified by quartiles, followed by TyG-1h (OR 2.298, 95% CI 1.248-4.234) and TyG-2h (OR 2.115, 95% CI 1.175-3.807). ROC curves suggested that TyG-1h and TyG-2h demonstrated superior diagnostic performance compared to TyG, with AUCs of 0.685, 0.679 and 0.673, respectively.

**Conclusions:**

The modified TyG indices exhibited strong effectiveness in identifying arterial stiffness in Chinese overweight or obese individuals.

## 1 Introduction

Insulin resistance (IR) is a condition characterized by the diminished ability of insulin to elicit its normal physiological effects in its target tissues, primarily muscle, adipose, and liver (1). IR is a significant contributing factor to arterial stiffness and the development of cardiovascular diseases (CVD) due to its role in promoting inflammation and subsequent endothelial damage (2).

It is widely recognized that the hyperinsulinemic-euglycemic glucose clamp serves as the definitive method for assessing IR, yet its practical use in clinical settings is constrained by its lengthy duration and significant expense (3). Alternatively, the homeostatic model assessment of IR (HOMA-IR), derived from fasting blood glucose (FBG) and insulin concentrations, is the most prevalent and validated indicator of IR in clinical practice (3). However, insulin levels in the blood are typically measured in the context of diabetes mellitus, making them impractical for general use. Consequently, various alternative markers of IR have been introduced recently, among which is the triglyceride-glucose (TyG) index (4).

The TyG index, a metric derived from fasting triglyceride (TG) and FBG concentrations, is strongly correlated with IR (5). Prior investigations have consistently shown a positive link between the TyG index and cardiovascular disease (CVD), as well as a heightened risk of arterial stiffness, as evaluated by brachial-ankle pulse wave velocity (baPWV) (4). Recent studies have further indicated the potential of the modified TyG indices, such as the triglyceride−glucose−waist circumference (TyG−WC) and the triglyceride−glucose−waist−height ratio (TyG−WHtR), as simple and cost-effective markers for identifying individuals who may be predisposed to cardiovascular events or metabolic disorders (6–9).

Obesity, a major health risk factor, is closely linked to the development of IR (10). However, it remains unclear whether the TyG index or its modified versions continue to serve as reliable predictors of arterial stiffness in overweight or obese individuals. Therefore, our study aims to investigate the correlation of the TyG index and TyG-related parameters with arterial stiffness in overweight or obese individuals, as well as to explore novel modified TyG indices that could aid in identifying subjects at high risk.

## 2 Methods

### 2.1 Study population

This retrospective study included patients admitted to the First Affiliated Hospital of Ningbo University from May 2021 to December 2023, primarily presenting with complaints of excessive weight gain. A total of 1,143 patients were selected based on specific inclusion criteria: aged between 18 and 75 years, had a body mass index (BMI) of 24 kg/m² or higher, had undergone an oral glucose tolerance test (OGTT), and had available data on peripheral arterial sclerosis. However, patients were excluded if they had a prior diagnosis of diabetes, were currently using lipid-lowering medications, had a history of coronary atherosclerotic heart disease or stroke, suffered from severe liver or kidney dysfunction, or presented with hematological disorders, chronic infectious diseases, or malignant tumors. The study adhered to the Helsinki Declaration and received approval from the Ethics Committee of The First Affiliated Hospital of Ningbo University (2024-158RS). And informed consent was waived owing to the retrospective nature of the study.

### 2.2 Demographic, medical, and laboratory data

For all patients enrolled in the study, demographic details such as sex, age, and prior medical history were collected. Trained nurses subsequently conducted anthropometric evaluations, adhering to standardized protocols, to measure height, weight, waist circumference (WC), systolic blood pressure (SBP), and diastolic blood pressure (DBP). The baPWV was measured via an arterial sclerosis detector (BP-203RPE III, Omron Corporation, Japan) according to the manufacturer’s instructions (11). An average baPWV ≥1400 cm/s of both sides was considered indicative of arterial stiffness (12).

Following an overnight fast, venous blood samples were obtained from the participants. The analysis included measurements of glycated haemoglobin (HbA1c), FBG, fasting insulin levels, triglyceride (TG), total cholesterol (TC), low density lipoprotein-cholesterol (LDL-C), high density lipoprotein-cholesterol (HDL-C), serum creatinine, uric acid, and routine blood tests. The OGTT was conducted according to standard procedures as described previously (13), and 1-hour and 2-hour post-load plasma glucose (PG) levels and insulin levels were recorded.

### 2.3 Calculations

BMI was derived by dividing an individual’s weight (in kilograms) by the square of their height (in meters). The HOMA-IR index was computed via the standard formula, which involves multiplying FBG (in mmol/L) by fasting insulin levels (in uIU/mL) and then dividing the result by 22.5 (13). For the calculation of the TyG index and its modified versions, the following formulas were applied (14, 15):


TyG = ln [fasting TG (mg/dL) × FBG (mg/dL)/2];

TyG‐WC = TyG × WC (cm);

TyG‐WHtR = TyG × WC (cm)/height (cm);

TyG‐1h = ln [fasting TG (mg/dL) × 1‐hour PG (mg/dL)/2];

TyG‐2h = ln [fasting TG (mg/dL) × 2‐hour PG (mg/dL)/2].

### 2.4 Statistical analysis

Patients were grouped based on baPWV to describe their basic characteristics. Independent-samples t-test was used to compare normally distributed continuous variables, presented as mean ± standard deviation (SD). Non-normally distributed continuous variables were expressed as median and interquartile range, and the Mann-Whitney U test was used. Categorical variables, expressed as frequencies (percentages), were compared via the chi-square test.

Multiple logistic regression analysis was performed to assess the odds ratios (ORs) for the risk of arterial stiffness as IR indicators increased, analyzed both as continuous variables and stratified by quartiles. The analyses were first conducted without adjustment, followed by adjustments for age and sex (Model 1), ad further adjusted for SBP, DBP, BMI, HbA1c, creatinine, uric acid, TG, HDL-C, and LDL-C (Model 2). The selection of confounders for inclusion in the multivariate model was guided by both the literature review and the results from univariate analyses. The variables were checked for multicollinearity, and none were found. Moreover, to further explore the dose−response relationship between IR indicators and baPWV, we performed a restricted cubic spline (RCS) analysis, adjusting for the same confounders as in Model 2. This analysis featured three knots positioned at the 10th, 50th, and 90th percentiles, with the median serving as the point of reference. Additionally, we conducted a receiver operating characteristic (ROC) curve analysis to assess the predictive capability of IR indicators, which were treated as continuous variables. Statistical significance was set at a P value of<0.05 (two-tailed). All data analyses were carried out via IBM SPSS Statistics for Windows (version 27.0) and R software (version 4.4.1).

## 3 Results

### 3.1 Basic characteristics

A total of 1143 overweight or obese individuals, comprising 409 (35.8%) males and 734 (64.2%) females, were included in this retrospective cross-sectional study. **Table 1** presents the baseline characteristics of the study population, stratified based on baPWV. The mean age of the individuals was 33.6 ± 9.7 years, and 321 individuals (28.1%) were identified as having arterial stiffness.

**Table 1:** Clinical characteristics of patients stratified by baPWV.

	baPWV<1400 (n = 822)	baPWV≥1400 (n = 321)	*P* value
Age, years	31.88 ± 8.72	38.08 ± 10.51	<0.001
Male, n (%)	250 (30.4)	159 (49.5)	<0.001
SBP, mmHg	131.49 ± 15.60	145.80 ± 18.12	<0.001
DBP, mmHg	79.04 ± 10.37	88.60 ± 12.84	<0.001
BMI, kg/m^2^	30.96 ± 3.92	31.62 ± 4.25	0.012
Waist Circumference, cm	96.57 ± 11.01	101.29 ± 11.07	<0.001
WHtR	0.59 ± 0.06	0.61 ± 0.06	<0.001
HbA1c, %	5.45 ± 0.53	5.75 ± 0.72	<0.001
Fasting blood glucose, mmol/L	5.31 ± 0.74	5.76 ± 1.15	<0.001
OGTT 1h-PPG	9.53 ± 2.57	11.14 ± 3.05	<0.001
OGTT 2h-PPG	7.83 ± 2.32	9.12 ± 3.16	<0.001
Fasting insulin, mU/L	18.77 (13.90)	20.85 (16.40)	0.005
HOMA-IR	4.40 (3.55)	5.07 (4.35)	<0.001
Triglycerides, mmol/L	1.43 (0.98)	1.90 (1.27)	<0.001
Total cholesterol, mmol/L	5.30 ± 1.03	5.54 ± 1.10	<0.001
HDL-C, mmol/L	1.22 ± 0.23	1.20 ± 0.25	0.161
LDL-C, mmol/L	3.45 ± 0.75	3.63 ± 0.80	<0.001
Uric acid, μmol/L	398.04 ± 99.93	418.05 ± 112.10	0.005
Creatinine, μmol/L	62.49 ± 12.89	65.73 ± 14.98	<0.001
baPWV	1168.82 ± 140.92	1553.45 ± 161.46	<0.001
TyG	8.77 ± 0.57	9.10 ± 0.59	<0.001
TyG-1h	9.32 ± 0.67	9.74 ± 0.65	<0.001
TyG-2h	9.12 ± 0.66	9.52 ± 0.70	<0.001
TyG-WC	848.21 ± 123.49	922.64 ± 124.39	<0.001
TyG-WHtR	5.16 ± 0.68	5.57 ± 0.71	<0.001

Data are expressed as mean ± standard deviation, median (interquartile range) or n (%). *P<* 0.05 was considered statistically significant.

Individuals with arterial stiffness were generally older and had higher levels of SBP, DBP, BMI, WC, and WHtR. These individuals also had elevated HbA1c levels and higher results on the OGTT, including FBG, 1-hour, and 2-hour PG levels. Additionally, other biochemical markers related to metabolic disorders, such as higher serum creatinine, uric acid, TG, TC, and LDL-C levels, were observed in the high baPWV group. They also exhibited significantly elevated levels of fasting insulin, HOMA-IR and TyG-related parameters, as compared to the other group (P< 0.05). However, no statistically significant difference was observed in the levels of HDL-C between the two groups.

### 3.2 Associations between TyG indices and arterial stiffness

**Figure 1:**
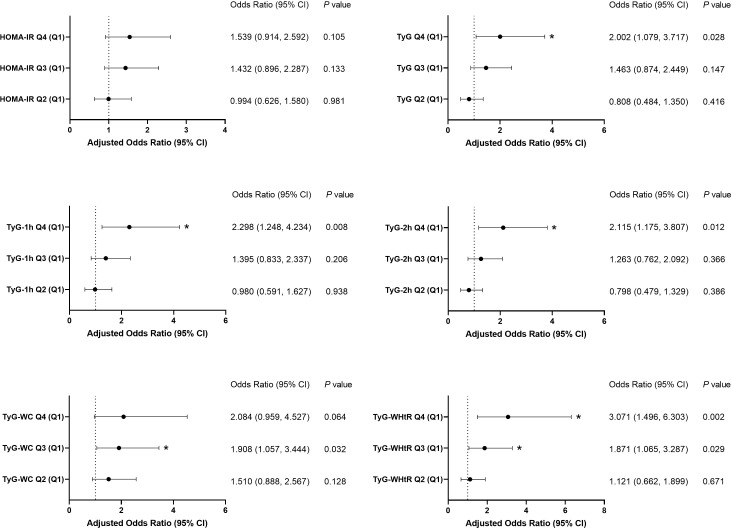
Association of the six parameters with arterial stiffness in Model 2. Each insulin-resistance indicator was stratified by quartiles. HOMA-IR quartiles: Q1 ≤ 3.11, 3.11< Q2 ≤ 4.60, 4.60< Q3 ≤6.78, Q4 > 6.78; TyG quartiles: Q1 ≤ 8.45, 8.45< Q2 ≤8.81, 8.81< Q3 ≤9.21, Q4 > 9.21; TyG-1h quartiles: Q1 ≤8.95, 8.95< Q2 ≤9.41, 9.41< Q3 ≤9.87, Q4 > 9.87; TyG-2h quartiles: Q1 ≤8.74, 8.74< Q2 ≤9.19, 9.19< Q3 ≤9.68, Q4 > 9.68; TyG-WC quartiles: Q1 ≤774.77, 774.77< Q2 ≤863.00, 863.00< Q3 ≤ 952.32, Q4 > 952.32; TyG-WHtR quartiles: Q1 ≤4.78, 4.78< Q2 ≤5.21, 5.21< Q3 ≤5.74, Q4 > 5.74. *P<0.05.

In the binary logistic regression model, all IR indicators, including HOMA-IR and TyG-related parameters, were independently correlated with an elevated risk of arterial stiffness after adjusting for sex and age (Model 1, **Table 2**). Furthermore, even after adjusting for additional confounders (SBP, DBP, BMI, HbA1c, creatinine, uric acid, TG, HDL-C, and LDL-C), all of the TyG-related parameters remained independent risk factors for elevated baPWV (Model 2, **Table 2**). When each IR indicator was stratified by quartiles, the maximum OR was observed for TyG-WHtR, which was as high as 3.071 (95% CI 1.496–6.303) in the maximal quartile (Q4) compared with the minimal quartile (Q1) (P = 0.002) in Model 2 (**Figure 1**). The OR was second highest for TyG-1h, reaching 2.298 (95% CI 1.248–4.234, P = 0.008), followed by TyG-2h with 2.115 (95% CI 1.175-3.807, P = 0.012).

**Table 2:** Association of the insulin resistance indicators with arterial stiffness.

	Model 1	*P* value	Model 2	*P* value
	OR (95% CI)		OR (95% CI)	
HOMA-IR	1.077 (1.040, 1.114)	<0.001	1.019 (0.983, 1.055)	0.304
TyG	2.016 (1.582, 2.570)	<0.001	1.944 (1.098, 3.444)	0.023
TyG-1h	1.970 (1.588, 2.445)	<0.001	1.832 (1.173, 2.860)	0.008
TyG-2h	1.869 (1.515, 2.306)	<0.001	1.715 (1.137, 2.585)	0.010
TyG-WC	1.004 (1.003, 1.006)	<0.001	1.005 (1.002, 1.007)	0.002
TyG-WHtR	2.054 (1.663, 2.537)	<0.001	2.871 (1.772, 4.652)	<0.001

Model 1: Adjusted for age and gender. Model 2: Adjusted for age, gender, SBP, DBP, BMI, HbA1c, Cr, UA, TG, HDL-C, and LDL-C.

**Figure 2:**
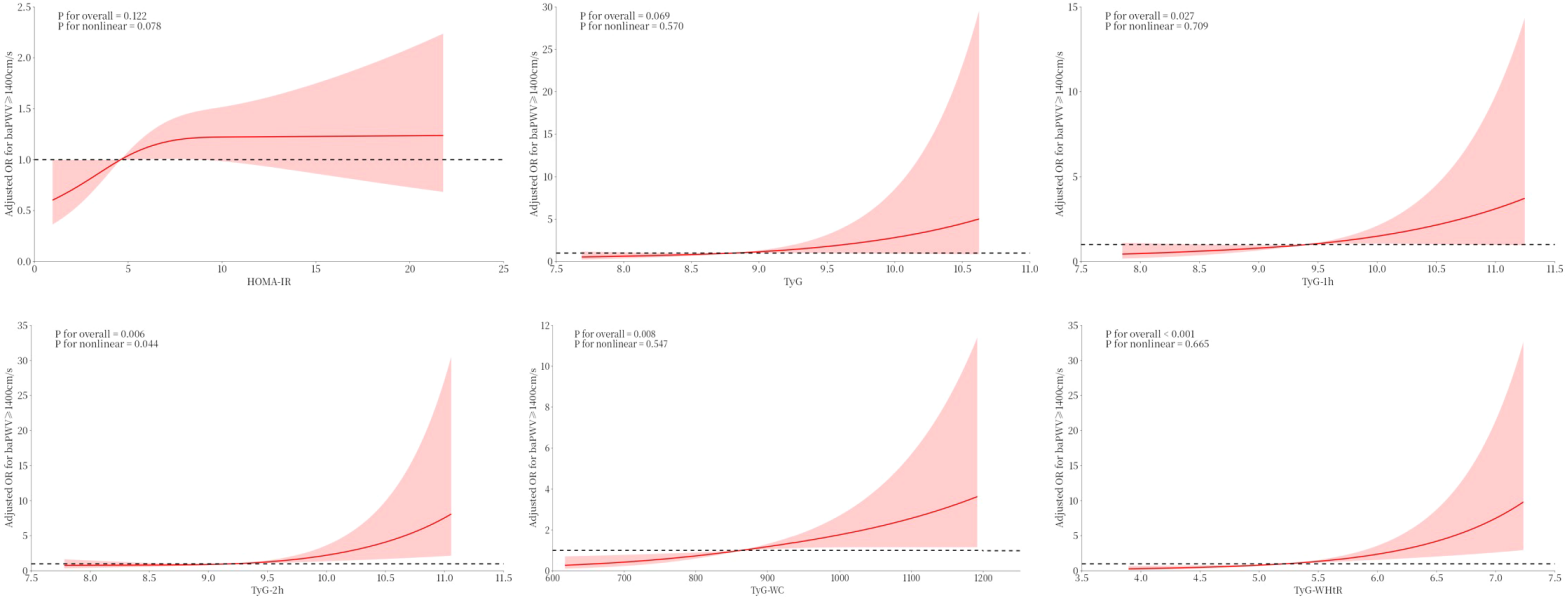
Restricted cubic splines analysis of IR indicators with arterial stiffness after adjustment for age, gender, SBP, DBP, BMI, HbA1c, Cr, UA, TG, HDL-C, and LDL-C. Data are shown as odd ratios (ORs, the solid lines) with 95% confidence intervals (CIs, the shaded regions).

By utilizing the RCS methodology, we crafted a flexible model to capture and visually represent the associations between various IR indicators and arterial stiffness. After adjusting for all confounders in Model 2, TyG-1h, TyG-WC and TyG-WHtR displayed linear correlations with arterial stiffness, whereas TyG-2h displayed a nonlinear correlation (**Figure 2**).

### 3.3 Predictive value analysis

**Figure 3:**
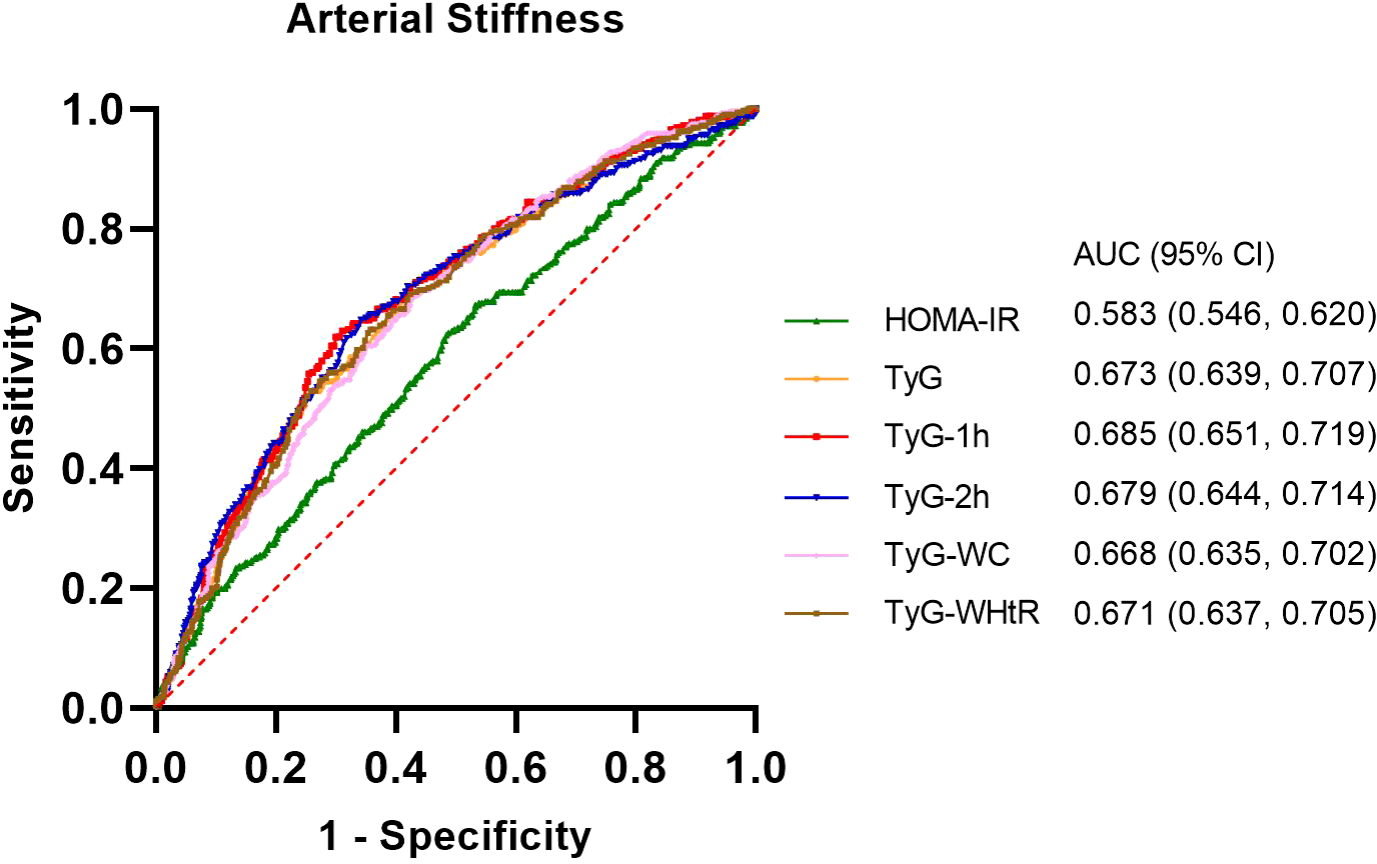
Receiver operating characteristic (ROC) curves and the area under the ROC curve (AUC) values of the six indicators (HOMA-IR, TyG, TyG-1h, TyG-2h, TyG-WC and TyG-WHtR) in diagnosing arterial stiffness.

All parameters reflecting IR were found to be statistically significant predictors of arterial stiffness (P< 0.05, **Figure 3**). Among these, the top three parameters with the highest AUC values were TyG-1h, TyG-2h, and TyG, with AUCs of 0.685, 0.679, and 0.673, respectively. The optimal cut-off value for TyG-1h in predicting arterial stiffness was determined to be 9.59, with a sensitivity of 62.0% and a specificity of 70.0%. The detailed results of this analysis are provided in **Supplementary Table 1**.

## 4 Discussion

In this study, a significant positive correlation between the risk of arterial stiffness and TyG-related indices, including TyG, TyG-1h, TyG-2h, TyG-WC and TyG-WHtR, was detected in overweight or obese individuals. When stratified by quartiles, TyG-WHtR displayed the best correlation, followed by TyG-1h and TyG-2h. ROC curve analyses demonstrated that all these parameters were significantly predictive of arterial stiffness. The modified indices, especially TyG-1h and TyG-2h, exhibited superior diagnostic capabilities compared with both HOMA-IR and the traditional TyG index in predicting high baPWV.

The TyG index, initially introduced as an alternative marker for IR, has been considered strongly correlated with IR determined through the hyperinsulinaemic-euglycaemic clamp method (14, 16). Multiple cross-sectional investigations have consistently reported a positive association between the TyG index and arterial stiffness, as measured by the baPWV (17–20). Upon further investigation, longitudinal studies have also yielded positive findings, reinforcing the association between the TyG index and the risk of arterial stiffness progression over time (21). The above studies involved different groups of individuals, including the general population and patients with hypertension or type 2 diabetes. Our work has expanded the scope of the research subjects. Regardless of various study subjects as mentioned above and the distinct definitions of arterial stiffness used in diverse research works, the results of the correlation between the TyG index and arterial stiffness were consistent with those of the above studies in multivariate and confounder-adjusted models.

Most recent investigations have revealed that the combination of the TyG index with obesity indicators, such as TyG-WC and TyG-WHtR, exhibits a significant and positive correlation with cardiovascular disease (CVD) and its associated mortality (22). Furthermore, both TyG-WC and TyG-WHtR demonstrated significantly greater diagnostic accuracy in predicting CVD and CVD-related mortality than did TyG (22). Other studies have suggested that TyG-WC and TyG- WHtR not only significantly correlate with the onset of hypertension (7) but also outperform the TyG index alone in predicting nonalcoholic or metabolic-associated fatty liver disease (23, 24). In accordance with most previous studies, our results revealed that TyG-WHtR displayed the best correlation with arterial stiffness in overweight or obese individuals. Obesity, independent of other cardiovascular risk factors, plays a pivotal role in both initiating and exacerbating cardiovascular disease, particularly when the distribution of body fat is considered (25). Consequently, incorporating obesity indicators into the TyG index offers a more precise assessment of insulin resistance compared to using either the HOMA-IR or the TyG index alone (26).

Despite the mounting research works surrounding the TyG index and its associated parameters, compelling suggestions among researchers to delve deeper into the potential clinical relevance of a postprandial TyG index have emerged (27). This inquiry stems from the fact that elevated postprandial levels of TG and glucose are recognized as metabolic abnormalities indicative of IR (27). Consequently, a heightened postprandial TyG index may serve as a marker for an increased predisposition to diabetes or cardiovascular events, underscoring its potential significance in clinical assessment and management. In this retrospective study, we replaced fasting glucose with 1-hour or 2-hour PG in the TyG formula to obtain a modified TyG index and compared the efficiency of arterial stiffness prediction among these parameters. Our study suggested that TyG-1h and TyG-2h remained independent risk factors for high baPWV after adjusting for confounders and achieved greater AUC values than did HOMA-IR and TyG, as well as TyG-WC and TyG-WHtR. A previous study involving subjects with different glucose tolerances indicated that the 1-hour PG level from the OGTT maintained an independent association with baPWV in a multiple regression analysis (28). Recently, the International Diabetes Federation issued a position statement highlighting the 1-hour PG as a crucial tool for diagnosing intermediate hyperglycemia and type 2 diabetes (29). This statement underscores the importance of the 1-hour PG in facilitating the early identification of individuals who are at heightened risk for abnormal glucose metabolism. With a combination of 1-hour PG and fasting TG, the novel modified TyG index, TyG-1h and TyG-2h, displayed better diagnostic efficacy for arterial stiffness than the TyG index and might be a potential marker for various metabolic disorders and related cardiovascular events. Moreover, future studies on a postprandial TyG index that integrates postprandial TG and PG levels would be an interesting topic to explore.

Insulin serves as a pivotal hormone that orchestrates cellular metabolism across various tissues in the human body. Insulin resistance arises when tissues become less responsive to insulin signaling, which is characterized by impaired glucose uptake and oxidation, diminished glycogen synthesis, and a weakened capacity to suppress lipid oxidation (30). This condition precipitates numerous metabolic shifts that contribute to the onset of cardiovascular disease (31). One such consequence is the disruption of glucose metabolism, leading to chronic hyperglycemia, which sparks oxidative stress and an inflammatory cascade, ultimately damaging cells (32). Furthermore, insulin resistance alters systemic lipid metabolism, fostering the development of dyslipidemia and contributing to atherosclerotic plaque formation (33). Obesity is correlated with an elevated risk of CVD, with central obesity specifically linked to insulin resistance (34). Nevertheless, the precise molecular pathway through which fat triggers insulin resistance remains unclear. Exploring practical and convenient measurements based on these mechanisms can aid in identifying overweight or obese individuals with elevated IR and high risks of arterial stiffness.

Obesity does not encompass a uniform group of individuals. Research has classified metabolic obesity phenotypes according to their metabolic dysfunction (35). Metabolically healthy obesity (MHO) typically refers to individuals with a BMI in the obese range but who do not meet the criteria for metabolic syndrome (MetS) or its individual components (36). MHO is associated with more favorable health outcomes compared to metabolically unhealthy obesity (MUO). MHO patients exhibit insulin sensitivity comparable to that of healthy individuals with normal weight and possess a lower amount of visceral fat, as well as reduced cardiovascular risks, in comparison to the majority of MUO patients (37). However, at present, there is a lack of reliable biomarkers that can differentiate between the two conditions. The IR indicators suggested in this study hold the promise of aiding in distinguishing MHO from MUO, as well as recognizing those with elevated risk for CVD. More large-scale prospective cohort studies are needed to confirm this in the future, and our results provide a foundation for further research.

Importantly, this study has several limitations that need to be considered. First, the cross-sectional design could not lead to a conclusion on a causal relationship between the IR indicators and arterial stiffness. Second, the absence of data on exercise patterns, dietary habits, and alcohol consumption of the participants constitutes a gap in our understanding, as these factors can influence circulating TG levels. In particular, the absence of data on smoking status, which is considered a conventional risk factor for arterial stiffness, might influence the reliability of the conclusion. Furthermore, as a retrospective real-world study, the 1-hour post load TG was not measured as a regular biochemical marker. Finally, the study’s sample population, being confined to a single healthcare center, might limit the generalizability of the findings to broader populations. To verify the deeper association between TyG-related parameters and arterial stiffness, further prospective studies should be conducted with a larger number of participants.

## 5 Conclusion

In conclusion, this study introduced two novel TyG parameters that combine the traditional TyG index and post-load plasma glucose from the OGTT. Our work demonstrated that the TyG index and the modified TyG indices, including TyG-1h, TyG-2h, TyG-WC and TyG-WHtR were independently associated with arterial stiffness measured through baPWV in overweight or obese individuals. The TyG-WHtR displayed the best correlation with arterial stiffness, followed by TyG-1h and TyG-2h. Furthermore, TyG-1h and TyG-2h emerged as the most reliable indicators for the early detection of elevated baPWV among the IR parameters. These outcomes have broadened the limited scope of available knowledge regarding the link between the TyG index and arterial stiffness, offering potential benefits for the early recognition of individuals at elevated risk for this condition. By enabling earlier detection, appropriate preventative measures can be implemented to mitigate the risk of cardiovascular disease.

## Data Availability

The raw data supporting the conclusions of this article will be made available by the authors, without undue reservation.

## References

[1] Lee SH Park SY Choi CS. Insulin resistance: from mechanisms to therapeutic strategies. Diabetes Metab J. (2022) 46:15–37. doi: 10.4093/dmj.2021.0280 34965646 PMC8831809

[2] Laakso M Kuusisto J. Insulin resistance and hyperglycaemia in cardiovascular disease development. Nat Rev Endocrinol. (2014) 10:293–302. doi: 10.1038/nrendo.2014.29 24663222

[3] Freeman AM Acevedo LA Pennings N. Insulin resistance. In: StatPearls. StatPearls Publishing, Treasure Island (FL (2023). 29939616

[4] Sánchez-Íñigo L Navarro-González D Fernández-Montero A Pastrana-Delgado J Martínez JA. The TyG index may predict the development of cardiovascular events. Eur J Clin Invest. (2016) 46:189–97. doi: 10.1111/eci.12583 26683265

[5] Du T Yuan G Zhang M Zhou X Sun X Yu X. Clinical usefulness of lipid ratios, visceral adiposity indicators, and the triglycerides and glucose index as risk markers of insulin resistance. Cardiovasc Diabetol. (2014) 13:146. doi: 10.1186/s12933-014-0146-3 25326814 PMC4209231

[6] Cui C Qi Y Song J Shang X Han T Han N. Comparison of triglyceride glucose index and modified triglyceride glucose indices in prediction of cardiovascular diseases in middle aged and older Chinese adults. Cardiovasc Diabetol. (2024) 23:185. doi: 10.1186/s12933-024-02278-z 38812015 PMC11138075

[7] Lee JH Heo SJ Kwon YJ. Sex-specific comparison between triglyceride glucose index and modified triglyceride glucose indices to predict new-onset hypertension in middle-aged and older adults. J Am Heart Assoc. (2023) 12:e030022. doi: 10.1161/JAHA.123.030022 37721166 PMC10547265

[8] Kim AH Son DH Lee YJ. Modified triglyceride-glucose index indices are reliable markers for predicting risk of metabolic dysfunction-associated fatty liver disease: a cross-sectional study. Front Endocrinol (Lausanne). (2024) 14:1308265. doi: 10.3389/fendo.2023.1308265 38317718 PMC10839046

[9] Park HM Han T Heo SJ Kwon YJ. Effectiveness of the triglyceride-glucose index and triglyceride-glucose-related indices in predicting cardiovascular disease in middle-aged and older adults: A prospective cohort study. J Clin Lipidol. (2024) 18:e70–9. doi: 10.1016/j.jacl.2023.11.006 38044202

[10] Barazzoni R Gortan Cappellari G Ragni M Nisoli E. Insulin resistance in obesity: an overview of fundamental alterations. Eat Weight Disord. (2018) 23:149–57. doi: 10.1007/s40519-018-0481-6 29397563

[11] Yamashina A Tomiyama H Takeda K Tsuda H Arai T Hirose K. Validity, reproducibility, and clinical significance of noninvasive brachial-ankle pulse wave velocity measurement. Hypertens Res. (2002) 25:359–64. doi: 10.1291/hypres.25.359 12135313

[12] Fan Y Wang Z Zhao X Wu S Chi H. Association of the visceral adiposity index with arterial stiffness in elderly Chinese population. Am J Med Sci. (2023) 365:279–85. doi: 10.1016/j.amjms.2022.10.010 36335991

[13] Stumvoll M Mitrakou A Pimenta W Jenssen T Yki-Järvinen H Van Haeften T. Use of the oral glucose tolerance test to assess insulin release and insulin sensitivity. Diabetes Care. (2000) 23:295–301. doi: 10.2337/diacare.23.3.295 10868854

[14] Simental-Mendía LE Rodríguez-Morán M Guerrero-Romero F. The product of fasting glucose and triglycerides as surrogate for identifying insulin resistance in apparently healthy subjects. Metab Syndr Relat Disord. (2008) 6:299–304. doi: 10.1089/met.2008.0034 19067533

[15] Yang C Song Y Wang X Yang Y Zhou Y Wang D. Association of hypertension with the triglyceride-glucose index and its associated indices in the Chinese population: A 6-year prospective cohort study. J Clin Hypertens (Greenwich). (2024) 26:53–62. doi: 10.1111/jch.14758 38133535 PMC10795092

[16] Mohd Nor NS Lee S Bacha F Tfayli H Arslanian S. Triglyceride glucose index as a surrogate measure of insulin sensitivity in obese adolescents with normoglycemia, prediabetes, and type 2 diabetes mellitus: comparison with the hyperinsulinemic-euglycemic clamp. Pediatr Diabetes. (2016) 17:458–65. doi: 10.1111/pedi.12303 26251318

[17] Lee SB Ahn CW Lee BK Kang S Nam JS You JH. Association between triglyceride glucose index and arterial stiffness in Korean adults. Cardiovasc Diabetol. (2018) 17:41. doi: 10.1186/s12933-018-0692-1 29562908 PMC5863385

[18] Liu F Ling Q Xie S Xu Y Liu M Hu Q. Association between triglyceride glucose index and arterial stiffness and coronary artery calcification: a systematic review and exposure-effect meta-analysis. Cardiovasc Diabetol. (2023) 22:111. doi: 10.1186/s12933-023-01819-2 37179288 PMC10183133

[19] Li M Zhan A Huang X Hu L Zhou W Wang T. Positive association between triglyceride glucose index and arterial stiffness in hypertensive patients: the China H-type Hypertension Registry Study. Cardiovasc Diabetol. (2020) 19:139. doi: 10.1186/s12933-020-01124-2 32948181 PMC7501677

[20] Wang S Shi J Peng Y Fang Q Mu Q Gu W. Stronger association of triglyceride glucose index than the HOMA-IR with arterial stiffness in patients with type 2 diabetes: a real-world single-centre study. Cardiovasc Diabetol. (2021) 20:82. doi: 10.1186/s12933-021-01274-x 33888131 PMC8063289

[21] Wu S Xu L Wu M Chen S Wang Y Tian Y. Association between triglyceride-glucose index and risk of arterial stiffness: a cohort study. Cardiovasc Diabetol. (2021) 20:146. doi: 10.1186/s12933-021-01342-2 34271940 PMC8285795

[22] Dang K Wang X Hu J Zhang Y Cheng L Qi X. The association between triglyceride-glucose index and its combination with obesity indicators and cardiovascular disease: NHANES 2003-2018. Cardiovasc Diabetol. (2024) 23:8. doi: 10.1186/s12933-023-02115-9 38184598 PMC10771672

[23] Song K Park G Lee HS Lee M Lee HI Choi HS. Comparison of the triglyceride glucose index and modified triglyceride glucose indices to predict nonalcoholic fatty liver disease in youths. J Pediatr. (2022) 242:79–85.e1. doi: 10.1016/j.jpeds.2021.11.042 34808224

[24] Xue Y Xu J Li M Gao Y. Potential screening indicators for early diagnosis of NAFLD/MAFLD and liver fibrosis: Triglyceride glucose index-related parameters. Front Endocrinol (Lausanne). (2022) 13:951689. doi: 10.3389/fendo.2022.951689 36120429 PMC9478620

[25] Lavie CJ Milani RV Ventura HO. Obesity and cardiovascular disease: risk factor, paradox, and impact of weight loss. J Am Coll Cardiol. (2009) 53:1925–32. doi: 10.1016/j.jacc.2008.12.068 19460605

[26] Lim J Kim J Koo SH Kwon GC. Comparison of triglyceride glucose index, and related parameters to predict insulin resistance in Korean adults: An analysis of the 2007-2010 Korean National Health and Nutrition Examination Survey. PloS One. (2019) 14:e0212963. doi: 10.1371/journal.pone.0212963 30845237 PMC6405083

[27] Tao LC Xu JN Wang TT Hua F Li JJ. Triglyceride-glucose index as a marker in cardiovascular diseases: landscape and limitations. Cardiovasc Diabetol. (2022) 21:68. doi: 10.1186/s12933-022-01511-x 35524263 PMC9078015

[28] Wang R Liu XL Jia XJ Liu Y Lu Q. One-hour post-load plasma glucose levels are associated with early arterial stiffness in subjects with different glucose tolerance. Diabetes Metab Syndr Obes. (2022) 15:1537–42. doi: 10.2147/DMSO.S368504 PMC9124057 35607609

[29] Bergman M Manco M Satman I Chan J Schmidt MI Sesti G. International Diabetes Federation Position Statement on the 1-hour post-load plasma glucose for the diagnosis of intermediate hyperglycaemia and type 2 diabetes. Diabetes Res Clin Pract. (2024) 209:111589. doi: 10.1016/j.diabres.2024.111589 38458916

[30] Janus A Szahidewicz-Krupska E Mazur G Doroszko A. Insulin resistance and endothelial dysfunction constitute a common therapeutic target in cardiometabolic disorders. Mediators Inflamm. (2016) 2016:3634948. doi: 10.1155/2016/3634948 27413253 PMC4931075

[31] Ormazabal V Nair S Elfeky O Aguayo C Salomon C Zuñiga FA. Association between insulin resistance and the development of cardiovascular disease. Cardiovasc Diabetol. (2018) 17:122. doi: 10.1186/s12933-018-0762-4 30170598 PMC6119242

[32] Bornfeldt KE Tabas I. Insulin resistance, hyperglycemia, and atherosclerosis. Cell Metab. (2011) 14:575–85. doi: 10.1016/j.cmet.2011.07.015 PMC3217209 22055501

[33] Goldberg IJ. Clinical review 124: Diabetic dyslipidemia: causes and consequences. J Clin Endocrinol Metab. (2001) 86:965–71. doi: 10.1210/jcem.86.3.7304 11238470

[34] Shulman GI. Ectopic fat in insulin resistance, dyslipidemia, and cardiometabolic disease [published correction appears in N Engl J Med. N Engl J Med. (2014) 371:1131–41. doi: 10.1056/NEJMra1011035 25229917

[35] Blüher M. Metabolically healthy obesity. Endocr Rev. (2020) 41:bnaa004. doi: 10.1210/endrev/bnaa004 32128581 PMC7098708

[36] Lavie CJ Laddu D Arena R Ortega FB Alpert MA Kushner RF. Healthy weight and obesity prevention: JACC health promotion series. J Am Coll Cardiol. (2018) 72:1506–31. doi: 10.1016/j.jacc.2018.08.1037 30236314

[37] Engin A. The definition and prevalence of obesity and metabolic syndrome: correlative clinical evaluation based on phenotypes. Adv Exp Med Biol. (2024) 1460:1–25. doi: 10.1007/978-3-031-63657-8_1 39287847

