# Supplementary material for: Correlations of the triglyceride−glucose index and modified indices with arterial stiffness in overweight or obese adults
Source: Front Endocrinol (Lausanne). 2024 Dec 17;15:1499120. doi: 10.3389/fendo.2024.1499120 (PMC11685072; doi:10.3389/fendo.2024.1499120)
Supplement: Supplementary file 1 [file Table1.docx]

Supplementary Material

# Supplementary Tables

**Supplement Table 1** The optimal cut-off values of the six parameters for increased arterial stiffness

|  | Cutoff value | Sensitivity | Specificity |
| --- | --- | --- | --- |
| HOMA-IR | 4.46 | 62.6% | 51.6% |
| TyG | 8.81 | 70.4% | 57.9% |
| TyG-1h | 9.59 | 62.0% | 70.0% |
| TyG-2h | 9.33 | 64.8% | 65.8% |
| TyG-WC | 863.29 | 68.5% | 57.5% |
| TyG-WHtR | 5.48 | 56.1% | 71.4% |
